# WNT7A promotes tumorigenesis of head and neck squamous cell carcinoma via activating FZD7/JAK1/STAT3 signaling

**DOI:** 10.1038/s41368-024-00279-y

**Published:** 2024-01-22

**Authors:** Qingling Huang, Yi Xiao, Ting Lan, Youguang Lu, Li Huang, Dali Zheng

**Affiliations:** 1 Department of Biochemistry and Molecular Biology, School of Basic Medical Sciences, Fujian Medical University, Fuzhou, China; 2 Fujian Key Laboratory of Oral Diseases, School and Hospital of Stomatology, Fujian Medical University, Fuzhou, China; 3 Department of Preventive Dentistry, School and Hospital of Stomatology, Fujian Medical University, Fuzhou, China; 4 Department of Dentistry, The First Affiliated Hospital of Fujian Medical University, Fuzhou, China

**Keywords:** Oral cancer, Extracellular signalling molecules

## Abstract

Wnt signaling are critical pathway involved in organ development, tumorigenesis, and cancer progression. WNT7A, a member of the Wnt family, remains poorly understood in terms of its role and the underlying molecular mechanisms it entails in head and neck squamous cell carcinoma (HNSCC). According to the Cancer Genome Atlas (TCGA), transcriptome sequencing data of HNSCC, the expression level of WNT7A in tumors was found to be higher than in adjacent normal tissues, which was validated using Real-time RT-PCR and immunohistochemistry. Unexpectedly, overexpression of WNT7A did not activate the canonical Wnt-β-catenin pathway in HNSCC. Instead, our findings suggested that WNT7A potentially activated the FZD7/JAK1/STAT3 signaling pathway, leading to enhanced cell proliferation, self-renewal, and resistance to apoptosis. Furthermore, in a patient-derived xenograft (PDX) tumor model, high expression of WNT7A and phosphorylated STAT3 was observed, which positively correlated with tumor progression. These findings underscore the significance of WNT7A in HNSCC progression and propose the targeting of key molecules within the FZD7/JAK1/STAT3 pathway as a promising strategy for precise treatment of HNSCC.

## Introduction

Head and neck squamous cell carcinoma (HNSCC) are a type of cancer that originates from the squamous cells lining the mucosal surfaces of the head and neck region, with an increasing incidence rate in recent years. It is estimated that as many as 380 000 new cases of HNSCC worldwide in 2020, with a trend towards earlier age of onset, particularly in high-incidence areas such as Asia and Africa.^1^ HNSCC usually exhibits an invasive growth pattern, frequently leading to regional lymph node or hematogenous metastases. Despite recent advances in treatment technologies, the unclear etiology and pathogenesis of HNSCC often result in limited therapeutic options and suboptimal outcomes, particularly in cases with local or distant metastases.^2^

The Wnt signaling pathway is a conserved signal transduction pathway in multicellular eukaryotes that exerts a wide range of biological effects. It plays a crucial role in various biological processes, such as cell growth, differentiation, proliferation, polarization, embryonic development, and stem cell self-renewal, and is involved in regulating most biological phenomena of life.^3,4^ Current research has established the crucial involvement of the Wnt signaling pathway in the onset and progression of numerous cancers.^5,6^ Aberrant activation of the Wnt signaling pathway affects the progress of several types of cells, enabling tumor cells to sustain and promote their stem cell phenotype, proliferation, and invasiveness. Among the identified cancers, over ten high-incidence malignancies result from abnormal activation of the Wnt signaling pathway, including colorectal cancer,^7^ lung cancer,^8^ breast cancer,^9^ and childhood acute lymphoblastic leukemia.^10^ Several studies have confirmed that Wnt signaling pathway imbalance can facilitate oral cancer development,^11^ and its abnormal activation can directly influence the prognosis of patients with oral cancer.^2^

The Wnt signaling pathway is a complex network involving 19 WNT ligand proteins, 10 receptor proteins, and multiple common or accessory proteins. The activation of this pathway, whether through canonical or non-canonical signaling, is always initiated by WNT ligand proteins. We analyzed the Cancer Genome Atlas (TCGA) (https://portal.gdc.cancer.gov/) transcriptome sequencing data of HNSCC and found that multiple WNT ligands were highly expressed in HNSCC, and the expression of WNT7A was significantly increased. WNT7A, a member of the WNT ligand family, plays diverse roles in different tumor types. In ovarian and endometrial cancers, it can promote cancer cell proliferation and induce cancer progression through the canonical Wnt-β-catenin pathway.^12^ However, in gastric carcinoma, WNT7A acts as a tumor suppressor and is independent of the canonical Wnt-β-catenin signaling.^13^ Recent studies have shown that WNT7A is upregulated in tongue squamous cell carcinoma (TSCC) and may be involved in the regulation of cell proliferation, migration, invasion, and epithelial-mesenchymal transition (EMT) in TSCC.^14^

Currently, the mechanism of WNT7A in tumorigenesis is a matter of debate. Some studies have reported that in non-small cell lung cancer cells, overexpression of WNT7A is accompanied by parallel changes in the JNK pathway, while phosphorylation of β-catenin (Thr41/Ser45, Ser552, Ser675, and Ser45) is not affected by WNT7A.^15^ Additionally, hyperactivation of the WNT signaling pathway and its associated factors is frequently observed in basal-like triple-negative breast cancer (TNBC).^16^ Furthermore, STAT3 is a crucial regulator of cancer stem cell function in various cancers, including TNBC.^17^ Therefore, some researchers suggest that the WNT and STAT3 pathways play critical roles in the initiation and metastasis of breast cancer.^18^ Our study aimed to explore the role of WNT ligands and the underlying relationship with STAT3 pathways in HNSCC.

## Results

### Elevated expression of WNT7A is associated with clinicopathological features of HNSCC

**Fig. 1:**
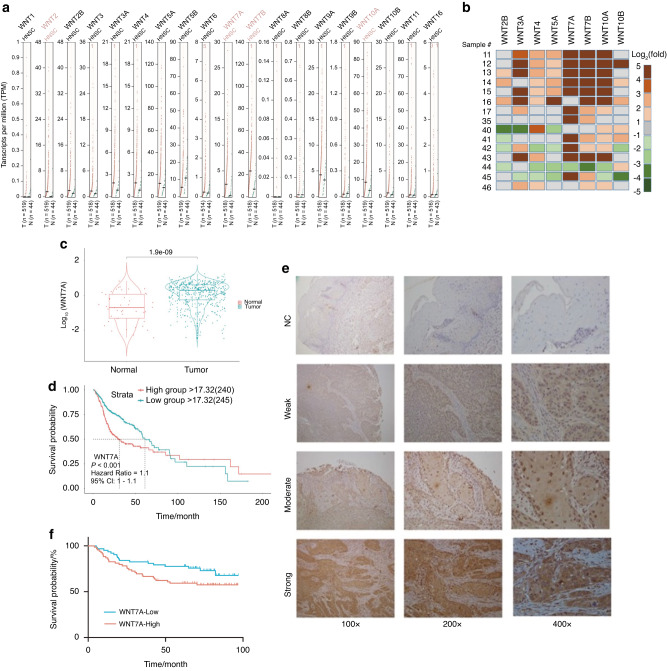
**Comprehensive analysis of WNT7A expression and its potential oncogenic role in HNSCC**. **a** Analysis of TCGA transcriptome sequencing data of HNSCC showed that several WNT ligands including WNT7A were up-regulated in HNSCC samples (shown in brown, *n* = 518) compared with para-cancerous (shown in green, *n* = 44). **b** Real-time RT-PCR analysis demonstrated that WNT7A exhibited significant upregulation in 11 out of 15 pairs of samples. The color change of the heatmap is based on the log_2_ (fold change). **c** Analysis of the TCGA database revealed that WNT7A was expressed at higher levels in HNSCC samples (shown in red, *n* = 496) compared to para-cancerous samples (shown in blue, *n* = 44). **d** Survival analysis showed the patients with high expression of WNT7A is associated with worse survival compared to their lower expression (*P* < 0.001). **e** Immunohistochemical evaluation of WNT7A staining in 137 pairs of HNSCC tissues and para-cancerous tissues was performed, and staining intensity was categorized into four grades: negative (score 0), weak positive (score 1), medium positive (score 2), and strong positive (score 3). Representative images of each staining grade are provided. **f** Overall survival analysis of 137 HNSCC patients indicated the high expression of WNT7A in cancer tissues (*n* = 75) was associated with poor survival compared with the patients with low expression (*n* = 62, *P* < 0.05)

To investigate the significance of the WNT family in the progression of HNSCC, we analyzed TCGA transcriptome sequencing data of HNSCC first. The data showed that mRNA expression levels of several WNT ligands were higher in tumors than para-cancerous (Fig. 1a). We verified these findings by performing real-time RT-PCR on 15 pairs of HNSCC tumors and their para-cancerous tissues. Several Wnt ligands were found to be up-regulated in tumors in these paired samples (Fig. 1b). Despite the heterogeneity of the tumors, the high expression of WNT7A remained consistent and significant in most cases. Mantel-Cox inspection and analysis showed that WNT7A was expressed at higher levels in HNSCC compared to para-cancerous tissues (*P* < 0.001) (Fig. 1c), and WNT7A expression level was negatively associated with patient overall survival rate (*P* < 0.001) (Fig. 1d).

We conducted immunohistochemical staining of WNT7A in a total of 137 HNSCC tissues, and representative images of each staining grade are presented in (Fig. 1e). Our analysis revealed that WNT7A was expressed in both HNSCC and para-cancerous tissues; however, its expression intensity was significantly higher in HNSCC tissues compared to para-cancerous tissues (Table 1). Specifically, WNT7A high expression was detected in 75 cases of HNSCC tissues, whereas only 11 cases of para-cancerous tissues were detected with WNT7A high expression (Table 1). Additionally, the total survival rate exhibited a statistically significant difference between the survival distributions of the WNT7A high and low expression groups (*P* < 0.05) (Fig. 1f). Notably, we observed a positive correlation between the WNT7A expression level and tumor differentiation (*P* < 0.05), and perineural invasion (*P* < 0.01). These findings suggest a potential oncogenic role of WNT7A in HNSCC.

**Table 1:** Immunohistochemical of WNT7A stain in 137 cases of HNSCC tissues

Characteristics	Cases	WNT7A(Low)	WNT7A(High)	*P* value
Cancer VS Normal				
Cancer	137	62	75	<0.001
Normal	137	126	11	***
Gender				
Male	93	38	55	0.132 9
Female	44	24	20	
Age				
Less than 60	87	42	45	0.348 8
60 and up	50	20	30	
Tumor Stages				
T1 and T2	58	28	30	0.604 6
T3 and T4	79	34	45	
Differentiation				
Well	83	44	39	0.023 7
Poorly or Moderately	54	18	36	*
WPOI-5				
Present	27	9	18	0.164 8
Not Identified	110	53	57	
Perineural Invasion				
Present	52	16	36	0.007 7
Not Identified	85	46	39	**
Extra nodal extension				
Present	21	9	12	0.810 4
Not Identified	116	53	63	
Lymph Node Metastasis				
N0	92	44	48	0.387 4
N1 and N2	45	18	27	

### WNT7A promotes HNSCC proliferation, self-renewal, and anti-apoptosis in vitro

**Fig. 2:**
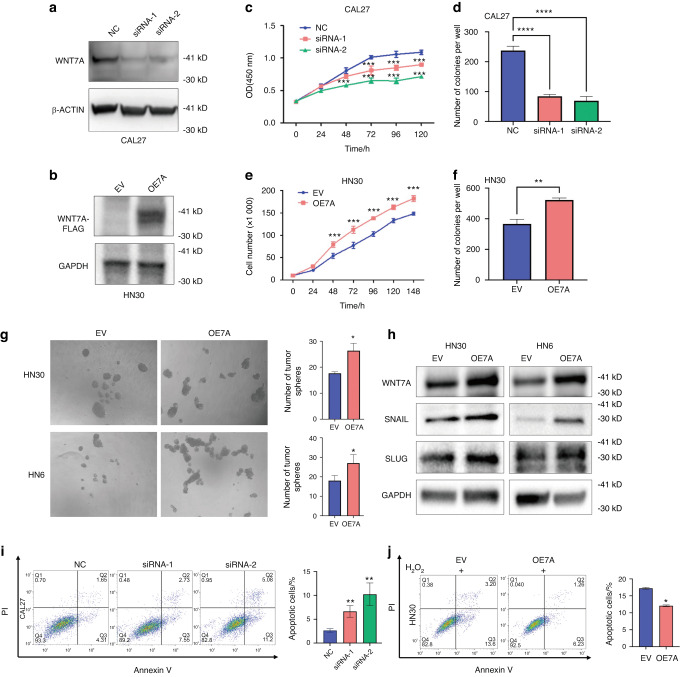
**Expression of WNT7A was positively associated with HNSCC cell proliferation, self-renewal, and anti-apoptosis**. **a**, **b** Western blot analysis validating the efficacy of WNT7A knockdown using siRNA interference (**a**), and overexpression through plasmid transfection in HNSCC cell lines (**b**). **c**–**f** Cell proliferation was assessed using cell counting method (**c**, **e**) and colony formation assays (**d**, **f**) after WNT7A knockdown (**c**, **d**) or overexpression (**e**, **f**). **g**, **h** Elevated WNT7A expression in HN30 and HN6 cells correlated with increased sphere formation (**g**) and stemness marker levels (**h**). **i** Flow cytometry analysis demonstrated that WNT7A knockdown substantially promoted apoptosis in CAL27 cells. **j** Conversely, WNT7A overexpression in HN30 cells led to a noteworthy reduction in apoptosis percentage compared to empty vector (EV) control, even under H_2_O_2_-induced stress. Data shown as mean ± SD (*n* = 3). *P < 0.05, **P < 0.01, ***P < 0.001, and ****P < 0.000 1

To investigate the potential oncogenic role of WNT7A in HNSCC, knockdown and overexpression experiments in HNSCC cell lines were conducted. Firstly, we analyzed the expression levels of WNT7A in several HNSCC cell lines. The expression of WNT7A in HN30 and CAL27 cell lines was higher than other cell lines, while the expression of WNT7A in HN6 cells was lower (Fig. S1A). Subsequently, we confirmed the efficiency of WNT7A siRNA knockdown in CAL27 cells (Fig. 2a) and ectopic expression following plasmid transfect in HN30 cells (Fig. 2b). Knockdown of WNT7A resulted in significant inhibition of cell growth (Fig. 2c and Fig. S1B), colony formation (Fig. 2d, and Fig. S1D, E) and tumor-sphere formation (Fig. S2A) in HNSCC cells. Consistently, overexpression of WNT7A significantly promoted cell growth and self-renewal in HNSCC cells (Fig. 2e–g and Fig. S1C, F). Stemness-related markers SNAIL^19,20^ and SLUG^21^ were also significantly regulated by WNT7A (Fig. 2h, and Fig. S2B, C).

In addition, flow cytometry analysis revealed that knockdown of WNT7A resulted in a significant increase in the apoptosis percentage of both CAL27 and HN30 cells (Fig. 2i, and Fig. S3A). Consistently, overexpression of WNT7A in HN30 and HN6 cells led to a significant decrease in the apoptosis percentage compared to the vector group, when induced by H_2_O_2_, a known apoptosis inducer for cell experiments.^22^ (Fig. 2j, and Fig. S3B). However, WNT7A was not associated with HNSCC cell mobility (Fig. S4A, and Fig. S4B). These findings further underscore the critical potential oncogenic role of WNT7A in HNSCC.

### WNT7A does not activate Wnt-β-catenin signaling in vitro

**Fig. 3:**
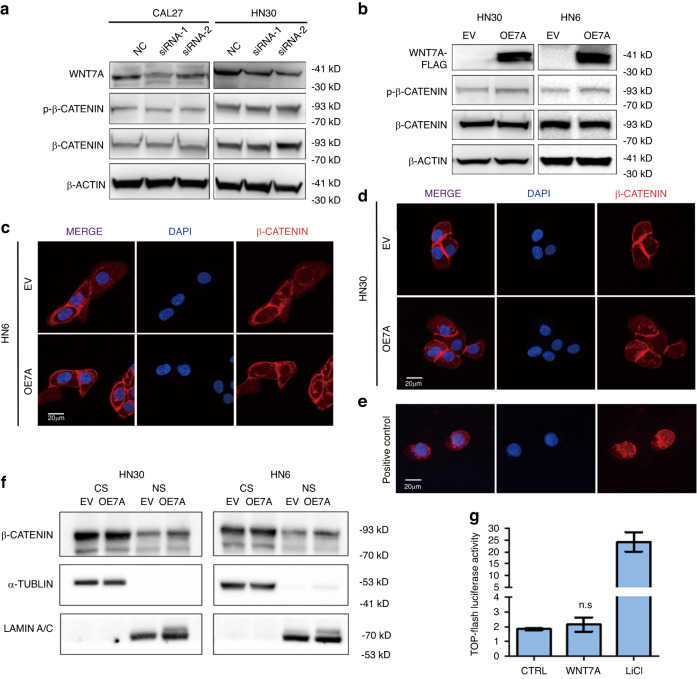
**WNT7A may not change the activation of the Wnt-β-catenin signaling pathway in HNSCC**. **a** Western blot analysis of CAL27 and HN30 cells with endogenous WNT7A knockdown showed no significant change in β-catenin protein levels or Thr41/Ser45 phosphorylation of β-catenin. **b** Overexpression of WNT7A did not change the Thr41/Ser45 phosphorylation of β-catenin. **c**, **d** Immunofluorescence shows β-catenin staining predominantly at the cell membrane and diffused in the cytoplasm, and weak nucleus β-catenin staining remains unchanged after overexpression of WNT7A. **e** Immunofluorescence showed clear nucleus β-catenin staining in the positive control group treated with 5 mmol/L LiCl. **f** Nucleus-cytoplasmic fractionation experiment showed no change in nucleus β-catenin translocation in cells of overexpressing WNT7A compared to the empty vector (EV) group. Cytoplasmic (CS), Nucleus (NS). **g** Luciferase assay showed no significant increase in TOP-Flash activity in cells treated with exogenous WNT7A compared to the empty vector (EV) group or positive control (LiCl). Data shown as mean ± SD (*n* = 3). *P < 0.05, **P < 0.01, ***P < 0.001, and ****P < 0.000 1

Next, we investigated the involved intracellular signaling pathways induced by WNT7A, initially focused on β-catenin which was a key component in the canonical WNT signaling pathway. Unexpectedly, the knockdown of endogenous WNT7A in CAL27 and HN30 cells did not result in the change of Thr41/Ser45 phosphorylation or a decrease in total protein level of β-catenin (Fig. 3a). Similarly, overexpression of WNT7A did not lead a change in Thr41/Ser45 phosphorylation of β-catenin and total β-catenin (Fig. 3b). Immunofluorescence staining revealed predominant presence of β-catenin at the cell membrane and dispersed distribution in the cytoplasm (Fig. 3c, d). Overexpression of WNT7A did not induce significant nucleus translocation of β-catenin in HNSCC cells (Fig. 3c, d). However, obvious nucleus β-catenin translocation was observed in the positive control group (Fig. 3e). Furthermore, in the nucleus-cytoplasmic separation experiment, we observed no significant nucleus translocation of β-catenin protein after WNT7A overexpression in cells (Fig. 3f).

To further assess the transcriptional activity of β-catenin following WNT7A stimulation, we conducted luciferase reporter assays in 293 T cells transfected with TOP-Flash plasmids. Consistently, the addition of exogenous WNT7A did not significantly increase luciferase activity compared to the control group. The luciferase activity was elevated obviously in the group treated with 5 mmol/L lithium chloride (LiCl), a known activator of canonical WNT signaling that inhibits glycogen synthetase kinase-3β^23^ (Fig. 3g). These findings collectively indicate that the WNT-β-catenin signaling pathway is unlikely to be involved in the effects of WNT7A in HNSCC.

### WNT7A upregulates the expression of STAT3 target genes

**Fig. 4:**
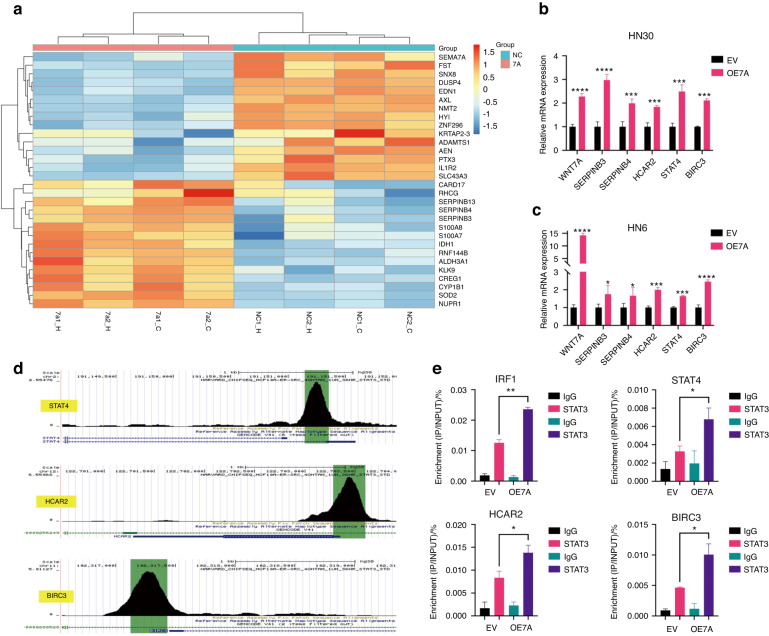
**WNT7A upregulated expression of STAT3 target genes in HNSCC cells**. **a** Heatmap of differentially expressed genes based on RNA-seq data. The top 30 deregulated genes were selected for further analysis. **b**, **c** Validation of the promote proliferation, self-renewal, and anti-apoptosis correlated genes by real-time RT-PCR. **d** Design of ChIP-qPCR primers by using Cistrome Data Browser. **e** ChIP-qPCR analysis was performed to examine the enrichment of STAT4, HCAR2, and BIRC3 after overexpression of WNT7A. The immunoprecipitation of the STAT3 was compared to that of the IgG antibody and IRF1 was a positive control in the analysis. Data shown as mean ± SD (*n* = 3). *P < 0.05, **P < 0.01, ***P < 0.001, and ****P < 0.000 1

We employed RNA-seq analysis combined with informatics tools and websites to examine the gene expression profiles associated with WNT7A expression (Fig. S5A). Initially, we identified genes that were potentially involved in proliferation, self-renewal and anti-apoptosis through Gene Ontology (GO) and Kyoto Encyclopedia of Genes and Genomes (KEGG) analysis.^24,25^ and the heatmap of top 30 deregulated genes was shown in Fig. 4a. To validate these findings, we performed real-time RT-qPCR experiments, confirming the differential expression of SERPINB3, SERPINB4, STAT4, HCAR2, and BIRC3 (Fig. 4b, c).

Furthermore, the ChEA3-ChIP-X Enrichment Analysis Version 3.1 tool.^26^ was employed to investigate the potential binding of transcription factors to the promoters of these identified genes. Remarkably, the analysis revealed that STAT3 exhibited potential binding to the promoters of several of these deregulated genes, suggesting a potential regulatory relationship between WNT7A and the STAT3 signaling pathway (Fig. S5B).

To investigate the potential direct target genes of STAT3 in HNSCC cells induced by WNT7A, we used CiiiDER2^27^ to predict transcription factor binding sites (Fig. S6A) and selected SERPINB3,^28^ SERPINB4,^29^ STAT4,^30^ HCAR2, and BIRC3^31,32^ for further verification, as these genes are related to tumor proliferation, self-renewal, and apoptosis based on GO analysis. We designed primers for ChIP-qPCR analysis by Cistrome Data Browser (Fig. 4d and Fig. S6B). Following the overexpression of WNT7A in HNSCC cells, we performed ChIP-qPCR analysis and used IRF1^33^ as a positive control, which is a known target gene of STAT3. After overexpression of WNT7A, the binding of STAT3 to the promoter region of STAT4, HCAR2, and BIRC3 was significantly enhanced (Fig. 4e). However, the binding to SERPINB3 and SERPINB4 (Fig. S6C, D) was not. These results indicate that WNT7A may induce STAT3-mediated noncanonical WNT signaling pathway activation in HNSCC.

### WNT7A activates FZD7/JAK1/STAT3 signaling in vitro

**Fig. 5:**
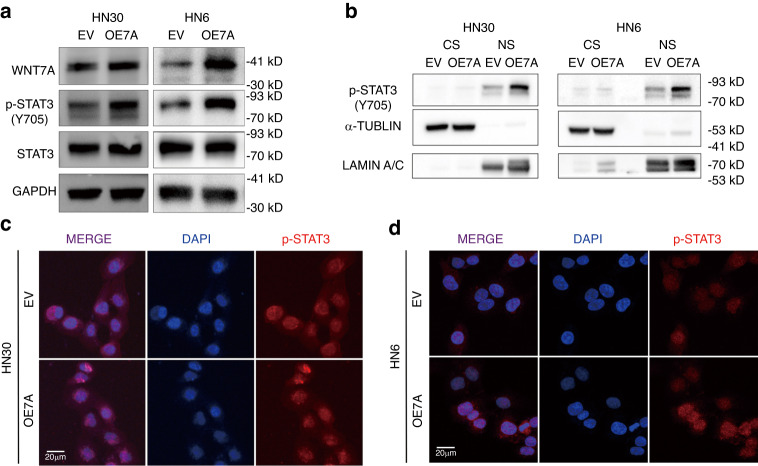
**WNT7A may activate STAT3 signaling pathway**. **a** Western blot analysis showed that overexpression of WNT7A increased pSTAT3 Y705 without altering total protein levels of STAT3 in HN30 and HN6 cells. **b** Nucleus-cytoplasmic separation experiment confirmed that nucleus pSTAT3 Y705 protein levels were significantly increased in the WNT7A overexpression group compared to the empty vector (EV) group. **c**, **d** Immunofluorescence showed that nucleus pSTAT3 staining was higher in the WNT7A overexpression group compared to the empty vector (EV) group. Cytoplasmic (CS), Nucleus (NS)

Y705 phosphorylation of STAT3 (pSTAT3 Y705) is a crucial step in the activation of STAT3 signaling pathway.^34^ So, we first verified this by overexpression WNT7A in HN30 and HN6 cells and found pSTAT3 Y705 expression increased without altering total protein levels of STAT3 (Fig. 5a). And nucleus pSTAT3 Y705 protein levels were significantly increased in the WNT7A overexpression group compared to the vector group by nucleus-cytoplasmic separation experiment (Fig. 5b). Immunofluorescence staining of pSTAT3 also confirmed these results, showing that pSTAT3 staining in two group presents in the nucleus, but pSTAT3 staining was stronger in the WNT7A overexpression group than in the vector group (Fig. 5c, d).

**Fig. 6:**
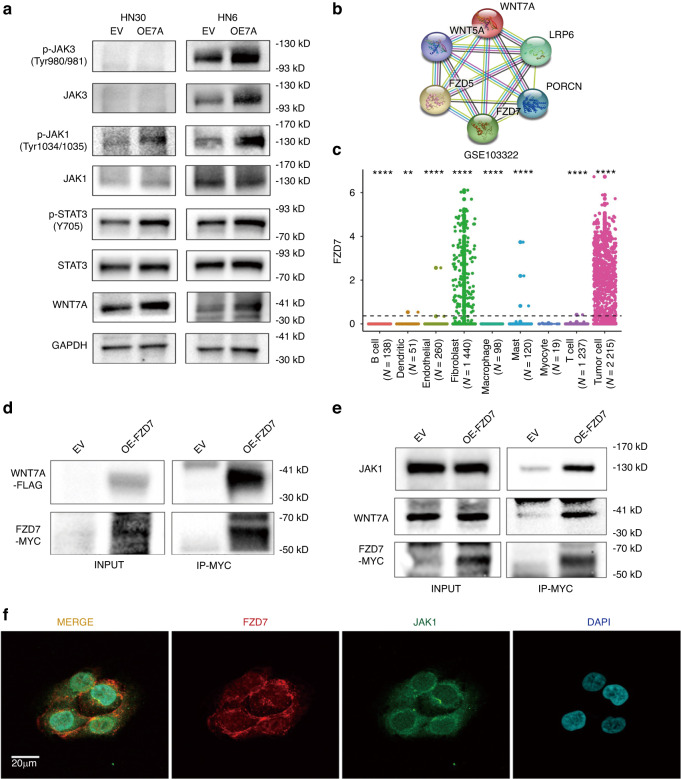
**WNT7A may activate STAT3 signaling pathway through FZD7/JAK1**. **a** Western blot analysis demonstrated that overexpression of WNT7A in HN30 and HN6 cells leaded to increased Tyr1034/1035 phosphorylation of JAK1, while total protein levels of JAK1 remain unchanged. **b** Protein network prediction using the STRING database identified FZD5 and FZD7 as potential receptor candidates for WNT7A. **c** Analysis of single-cell sequencing data (GSE103322) revealed higher enrichment of FZD7 in cancer cells. **d**, **e** Co-immunoprecipitation results confirm interactions between WNT7A, FZD7, and JAK1. **f** Co-localization assay by staining FZD7 (red), JAK1 (green), and nucleus (DAPI)

To further explore the underlying mechanism by which WNT7A regulates STAT3 phosphorylation, we examined the expression levels of upstream factors known to induce STAT3 activation, including JAK1, JAK2, and JAK3. Interestingly, we observed a significant increase in phosphorylated JAK1 levels after overexpressing WNT7A, while the expression of JAK2 was undetectable in HNSCC cell lines. Additionally, the expression of total JAK3 was altered too (Fig. 6a).

Considering that WNT7A acts as a secreted protein, we sought to determine its receptor target. According to the results of protein network prediction using the STRING (https://string-db.org/) database, FZD5 and FZD7 were considered as potential receptor candidates for WNT7A (Fig. 6b). Further analysis of single-cell sequencing data (GSE103322) revealed higher enrichment of FZD7 in cancer cells compared to FZD5 (Fig. 6c, and Fig. S7A, B). We further analyzed if WNT7A activated the JAK1/STAT3 signaling pathway through FZD7, and CoIP results showed FZD7, could bind with WNT7A and JAK1, but not JAK3 (Fig. 6d, e, and Fig. S7C). Immunofluorescence staining assay showed that FZD7 and JAK1 did colocalize together in cells (Fig. 6f). These results suggest that WNT7A can activate the JAK1/STAT3 signaling pathway through FZD7 in HNSCC, which has not been reported previously.

### TPCA inhibits HNSCC proliferation, self-renewal, and anti-apoptosis induced by STAT3 signaling pathway activation

**Fig. 7:**
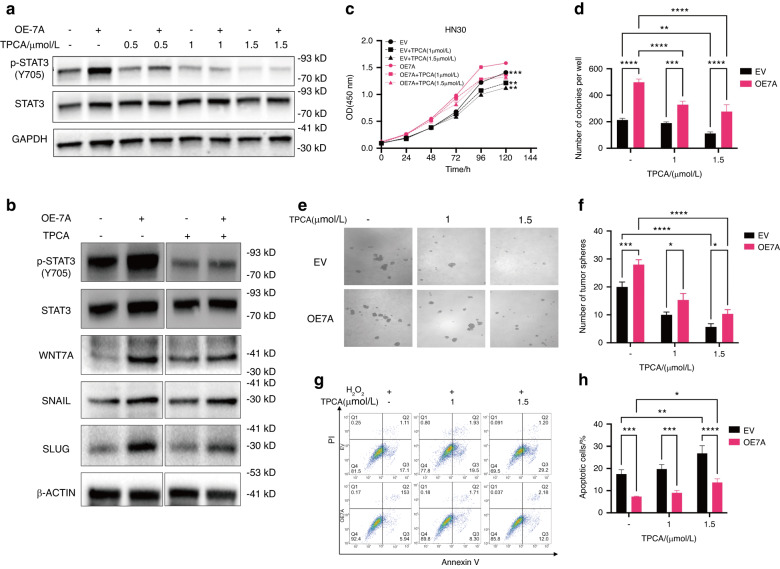
**TPCA inhibited WNT7A-induced activation of STAT3 signaling and proliferation, self-renewal, and anti-apoptosis in HNSCC cells**. **a** Western blot analysis showed the accumulation of pSTAT3 decreased with increasing TPCA concentration (0 μmol/L, 0.5 μmol/L, 1 μmol/L, 2 μmol/L), while the expression of pSTAT3 remains consistently higher in the WNT7A overexpression group. **b** Elevated WNT7A expression increased stemness marker levels but decreased after TPCA treatment. **c** CCK-8 assay showed that TPCA treatment decreased the cell proliferation promoted by WNT7A overexpression in HN30 cells. **d** Colony formation assay showed that TPCA treatment impaired the colony formation ability of HN30 cells. **e**, **f** Tumor sphere formation assay indicated that TPCA treatment impaired the sphere formation ability of HN30 cells. **g**, **h** Flow cytometry analysis shows that TPCA treatment increased the apoptotic percentage of HN30 cells. However, the WNT7A overexpression group is still lower than the empty vector (EV) group. Data shown as mean ± SD (*n* = 3). *P < 0.05, **P < 0.01, ***P < 0.001, and ****P < 0.000 1

To confirm whether WNT7A-induced activation of JAK1/STAT3 signaling directly affects HNSCC cell proliferation, self-renewal, and apoptosis, we used TPCA.^35^ to inhibit pSTAT3 Y705 in HN30 and HN6 cells. TPCA treatment abolished the accumulation of pSTAT3 Y705 stimulated by WNT7A overexpression in dose-dependent manner (Fig. 7a). And FZD7 overexpression also increased pSTAT3 Y705 levels, the phosphorylation level of STAT3 decreased after TPCA treatment (Fig. S7D). The expression of stemness-related markers in SNAIL and SLUG were also significantly decreased following treatment by TPCA (Fig. 7b). These results further confirmed that WNT7A can activate the JAK1/STAT3 signaling pathway.

The impact of WNT7A overexpression on cell growth was profound in both HN30 and HN6 cell lines. However, this stimulatory effect on cell growth was notably attenuated upon treatment with either 1 μmol/L or 1.5 μmol/L TPCA (Fig. 7c, and Fig. S8A). Remarkably, similar trends were observed in terms of colony formation, self-renewal, and anti-apoptosis ability of HNSCC cells (Fig. 7d–h, and Fig. S8B). These findings collectively underscore the potential therapeutic significance of targeting WNT7A and inhibiting pSTAT3 Y705 for innovative strategies in HNSCC treatment.

### WNT7A and pSTAT3 are positively associated with proliferation and anti-apoptosis in HNSCC PDX models

**Fig. 8:**
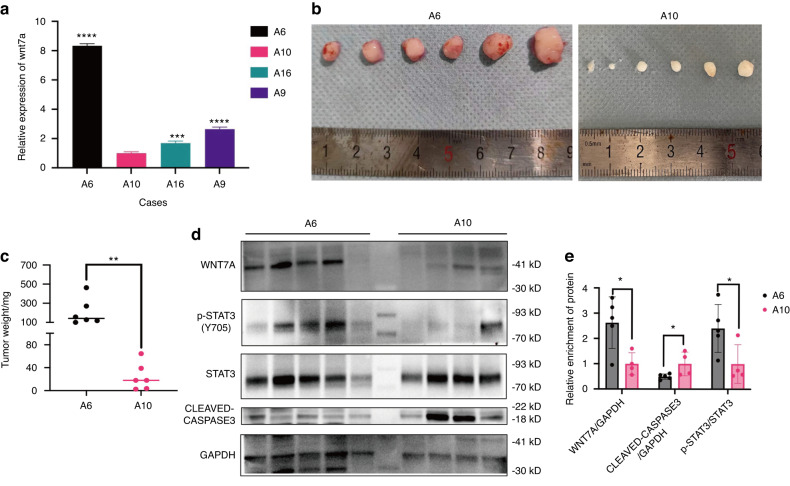
**WNT7A and pSTAT3 were associated with proliferation and anti-apoptosis in vivo**. **a** Expression profiling of WNT7A was conducted in four cases of second-generation PDX, and based on mRNA expression levels, A6 and A10 were selected for further analysis as A10 exhibited lower expression of WNT7A compared to A6 in the second generation. **b**, **c** A comparison was made between the tumor size (**b**) and weight (**c**) of A6 (*n* = 6) and A10 (*n* = 6). A10 displayed a smaller tumor volume and lower tumor weight when compared to A6. **d**, **e** Western blot analysis of proteins extracted from 5 mice with A6 PDX and 4 mice with A10 PDX. It was observed that WNT7A expression was positively correlated with the pSTAT3 Y705, indicating potential activation of the STAT3 signaling pathway. Additionally, there was a negative correlation between WNT7A expression and cleaved-caspase 3 levels. *P < 0.05, **P < 0.01, ***P < 0.001, and ****P < 0.000 1

According to our studies above in vitro, patient-derived xenograft (PDX) models were established using four clinical HNSCC samples to validate our findings. Expression profiling of the first generation of PDX tumors revealed variations in WNT7A expression among tumors from different patients, consistent with clinical observations (Fig. 8a). Based on these results, we selected two cases, A6 and A10, for further investigation. We observed a positive correlation between tumor size (Fig. 8b and Fig. S9A) and weight (Fig. 8c) with the levels of WNT7A expression and Y705 phosphorylation of STAT3 (Fig. 8d, e). Meanwhile, A10, which exhibited lower expression of WNT7A, displayed higher expression of cleaved Caspase3 compared to A6 (Fig. 8d, e). Cleaved Caspase3 is a marker of apoptotic cell death,^36^ and its levels were consistent with our in vitro findings. These findings suggest that WNT7A plays a crucial role in promoting HNSCC tumorigenesis and may serve as a potential therapeutic target for this aggressive malignancy.

## Discussion

The upregulation of WNT7A has been previously reported in different types of cancer, including colorectal cancer,^37^ lung cancer,^38^ and ovarian cancer,^12^ suggesting that WNT7A may be a common oncogenic factor in cancer. Our study further confirms that WNT7A is overexpressed in HNSCC, indicating its potential oncogenic role in this cancer as well. The correlation between WNT7A expression and HNSCC cell proliferation suggests that WNT7A may be involved in stemness and anti-apoptosis of HNSCC cells in our study. The positive correlation between WNT7A expression and survival rate also suggests that WNT7A may be a prognostic marker for HNSCC.

Previous studies have shown that WNT7A can promote tumor cell proliferation, migration, and invasion, as well as resistance to chemotherapy and radiotherapy through the Wnt-β-catenin pathway.^39,40^ However, for the first time, we found that after overexpression of WNT7A, the phosphorylation level of β-catenin Thr41/Ser45 was not decreased, and the total protein level and subcellular location of β-catenin were not affected. Based on previous studies, it has been suggested that WNT7A may have multiple domains that can bind to receptor proteins. However, some domains may not induce secondary effects or functional changes,^41^ which could be a reason why WNT7A did not activate the Wnt-β-catenin pathway in HNSCC. WNT7A has been found to bind to various receptor proteins in different tumor types, but the expression levels of these receptors may differ between HNSCC and other tumors.^42–44^ Additionally, there is a potential for secondary cascades. A study has shown that WNT can elevate galectin-3 expression, leading to its interaction with STAT3 and subsequent activation.^45^

Understanding the mechanism of malignancies will facilitate the identification of therapeutic and prognostic factors, thereby improving the efficiency of treatment for HNSCC patients.^46^ Our findings suggest that WNT7A may activate a previously undescribed STAT3-mediated noncanonical WNT pathway in HNSCC. The STAT3 signaling pathway has been extensively studied in tumor research due to its critical role in promoting tumorigenesis^47^. Numerous studies have reported that aberrant activation of STAT3 signaling promotes tumor growth,^46^ invasion, metastasis,^48^ and resistance to therapy.^49^ The JAK/STAT and Wnt-β-catenin pathways have been identified as pivotal players in tumorigenesis. Notably, research has demonstrated a direct interaction between STAT3 and β-catenin in the nucleus,^50^ with STAT3 positively regulating β-catenin’s transcriptional activity. Despite the lack of activation of the WNT-β-catenin pathway by WNT7A in HNSCC, our results indicate that WNT7A serves as the initiating factor for the STAT3 pathway. Additionally, being a secreted protein, WNT7A triggers a complex intracellular cascade through interaction with a receptor protein. Despite limited exploration of receptor expression in HNSCC, our investigation unveils a pivotal insight. Leveraging informatics tools and single-cell sequencing data, we demonstrate that WNT7A engages FZD7 to activate the JAK1/STAT3 signaling pathway in HNSCC. Notably, while STAT3 was previously proposed as a therapeutic target for HNSCC,^51^ our findings suggest a novel avenue for intervention upstream through WNT7A.

Taken together, our results suggest that WNT7A may play a critical role in the development and progression of HNSCC. The upregulation of WNT7A has been reported in various cancers, including HNSCC,^14^ indicating its potential role as an oncogenic factor in different types of cancer. Our study found that WNT7A expression is correlated with tumor growth and survival rate in HNSCC, suggesting its potential use as a prognostic marker. While previous studies have shown that WNT7A promotes tumor progression through the Wnt-β-catenin pathway,^39,40^ our study found that it activates a previously undescribed STAT3-mediated noncanonical Wnt pathway in HNSCC. Overall, our findings suggest that targeting WNT7A could be a potential therapeutic approach to inhibit STAT3-mediated signaling and HNSCC progression. Overall, the findings from this study provide valuable insights into the complex signaling pathways involved in HNSCC and lay a foundation for developing a new target combination therapy.

## Materials and methods

### Patients and samples

One hundred thirty-seven tumors and matching normal adjacent tissues were obtained from HNSCC patients at the First Affiliated Hospital of Fujian Medical University. The collection and use of HNSCC tissue samples were approved by the Institutional Ethics Committee of the First Affiliated Hospital of Fujian Medical University (China), and informed consent for publishing data relating to individual participants was obtained from the participants or their legal guardians.

### RNA sequencing and data analysis

RNA-seq results were performed using build GRCh38/hg38 as the Homo sapiens reference genome. Differentially expressed genes (DEGs) were identified using a filtered dataset quantified according to gene-level expression. To determine whether a set of genes showed statistically significant and/or concordant differences between two biological states, such as overexpression WNT7A versus vector group, DEGs were selected with a *p*-value < 0.01, false discovery rate (FDR) < 0.05, and fold change (FC) ≥ 1.5.

### Real-time RT-PCR

Total RNA was extracted from primary HNSCC tissues and cells using TRIzol reagent (Invitrogen), and cDNA was synthesized with the Prime Script RT Reagent Kit (TaKaRa). Real-time RT-PCR analyses were conducted using Real SYBR Mixture (CoWin Bioscience, China) on a Lightcycler 480 II instrument (Roche Applied Science), with GAPDH as an internal control. Table S2 shows the primers used for Real-time RT-PCR.

### Online cancer database analysis

The TCGA-HNSCC dataset containing gene expression and clinical data from 540 patients with HNSCC was obtained. Raw gene expression data was processed and normalized using the R Bioconductor package ‘limma’ and the ‘voom’ function. WNT7A expression levels were compared between tumor and normal samples using a moderated *t*-test. Survival analysis was conducted using Cox proportional hazards regression, with patients stratified into high and low-expression groups based on the median expression level of WNT7A.

### Immunohistochemical staining

WNT7A protein expression in HNSCC tissue was analyzed using immunohistochemical (IHC) staining on a tissue array. The tissue microarray chips contained a total of 137 samples (137 HNSCC and 137 para-cancerous) with follow-up data obtained from the affiliated hospital. All patient information was obtained and used following approved protocols from the institutional review boards of the participating institutions. Specific experimental methods referenced article published before.^52^ WNT7A expression was calculated as the product of the proportion score (%) multiplied by the staining intensity score (0–3). The proportion score represented the percentage of positive cells, while the intensity score represented the average intensity of staining (0: no staining, 1: yellow, 2: clay bank, and 3: tawny).

### Cell lines, primary cell preparation, and culture conditions

The human HNSCC cell lines, CAL27, HN30, HN6, FADU, and human embryonic kidney cell line, 293 T were cultured in DMEM high glucose media (SH30022.01, cytiva, China) with 10% fetal bovine serum (P30-3302, PAN-Biotech, German). All cells were cultured in a humidified incubator (Forma™ 351, Thermo Fisher) at 37 °C with 5% CO_2_.

### Plasmid design and transfection

We designed and synthesized siRNAs targeting WNT7A (Gene Pharma, Shanghai, China), whose specific sequences are shown in Table S1. CAL27 and HN30 cells were transfected with the siRNAs according to the instruction manual for Lipofectamine RNAiMAX (Invitrogen, Catalog # 13778150). The overexpression of WNT7A in HN6 and HN30 cells was performed using the pENTER plasmids, and a pair of primers was designed and synthesized. The primers were used to amplify the complete coding sequence (CDS) of the WNT7A gene (NM_004625) via PCR. The overexpression of FZD7(NM_003507) in HN6 and HN30 cells was performed using the pcDNA 3.1 plasmids. Transfection was performed following the instructions for Lipofectamine 3000 (Invitrogen, Catalog # L3000015).

### Cell growth, colony formation, sphere formation and cell apoptosis assay

Quantified cell growth using the Cell Counting Kit-8 Assay Kit (CK04, Dojindo, Japan). For the colony formation assay, cells were seeded into each well of a six-well plate and maintained in a medium containing 10% FBS for 14 days. The colonies were fixed with methanol and stained with 0.1% crystal violet. The clones containing at least 50 cells were counted using an inverted microscope. For the sphere formation assay, cells were seeded into each well of an Ultra-Low Attachment Surface 96 well plate (7007, Corning, USA) and maintained in DMEM/F12 (Gibco) + 1%B27 (Invitrogen, USA) + 20 ng/mL hFGF + 20 ng/mL hEGF for 14 days. We detected cell apoptosis by flow cytometry according to the manufacturer’s instructions of the Annexin V-FITC Apoptosis Detection Kit (F6012L, Bioscience, China).

### Protein extraction and Western blot assay

We extracted total cellular proteins using RIPA (P0013B, Beyotime, China), and lysate protein concentrations were quantified with a Bicinchoninic Acid Protein Assay (BCA) Kit (P1102, Beyotime, China). We used standard western blot assays to measure protein expression, and antibodies used to determine the indicated protein are shown in Table S4. The blots were visualized using a chemiluminescence detection system (ChemiDoc XRS+, Bio-Rad, USA).

### Nucleus-cytoplasmic separation assay

We washed cells with phosphate-buffered saline (PBS) and scraped off the cells with a cell scraper, and centrifuge to collect cells. The nucleus and cytoplasmic protein extract was performed following the instructions for the Nucleus Protein Extraction Kit (R0050, Solarbio, China). The nucleus and cytoplasmic proteins were then quantified using a BCA protein assay kit. Then standard western blot assays to measure protein expression.

### Dual luciferase reporter assay

To evaluate the transcriptional activity of the target, we employed the reporter construct pGL4.47[Luc2P/SIE/Hygro] (SIE-pGL4.47, Promega, Madison, WI), which harbors five copies of SIE, driving the luciferase reporter gene luc2P expression. The plasmids, as indicated, were transiently transfected into 293 T cells. To ensure a consistent total amount of transfected DNA in each well throughout all experiments, pRL-TKRenilla luciferase plasmid (Promega) was used. We transfected exogenous WNT7A into 293 T cells and collected the culture supernatants of each cell. After validating the expression of WNT7A in the culture supernatant by Western Blot with a labeled antibody (Flag), we added the culture supernatant to 293 T cells transfected with TOP-Flash plasmids. We also added the LiCl (310468, Sigma-Aldrich, Germany) to 293 T cells transfected with TOP-Flash plasmids as a positive control. Subsequently, the cells were harvested and their luciferase activity was assessed using the SpectraMax iD3 (USA).

### Immunofluorescence

The fixed cells were premobilized and blocked with 0.3% Triton X 100 (1139, BioFroxx, China) or 3% normal fetal bovine serum (4240, BioFroxx, China) in 0.01 mol/L PBS for 30 min at room temperature (RT). This was followed by overnight incubation with the designated antibodies at 4 °C. On the following day, the cells were incubated with fluorescein isothiocyanate conjugated antibody (Table S4 shows the antibodies used for Immunofluorescence). DAPI (C1005, Beyotime, China) was used to counterstain the cell nucleus. Subsequently, the cells were washed, mounted, and examined using a laser scanning confocal microscope (Leica Microsystems GmbH, Mannheim, Germany).

### GSE103322 single cell sequencing data analysis

According to the author’s notes, compare the expression level of WNT7A, FZD5, and FZD7 in different cell subclasses, the dotted line is the average expression of all cells, each subclass and the average expression are compared by Wilcox test, and the significance is marked in the figure.^53^

### Co-immunoprecipitation (Co-IP) assay

293 T were cultured in 100-mm dishes and transiently transfected with PC.DNA.3.1-vector or PC.DNA.3.1-FZD7-Myc-tag plasmids. At 80%–90% confluence, the cells were washed three times with ice-cold PBS and lysed on ice for 30 min using immunoprecipitation (IP) lysis buffer. The lysates were clarified by centrifugation at 14 000 × *g* for 10 min and incubated with 20 μL of Anti-c-Myc Magnetic Beads (HY-K0206, MedChemExpress, USA) overnight at 4 °C with rotation. Then washed six times with lysis buffer. The pelleted beads were resuspended in 30 μl loading buffer for SDS-PAGE followed by western blotting using the indicated antibodies.

### Chromatin immunoprecipitation (ChIP-qPCR)

ChIP assays were conducted using an anti-STAT3 antibody (Table S4 shows the antibodies used for ChIP) and Protein G Magnetic Beads (HY-K0204, MCE, USA) to pull down DNA fragments bound to STAT3. Specific primers were used to detect the binding of STAT3 to target genes after the DNA was purified and subjected to qPCR analysis. The input DNA was used as a control, and the enrichment of the target gene promoter was calculated as the ratio of immunoprecipitated DNA to input DNA. The immunoprecipitation of the STAT3 was compared to that of the IgG antibody (A00002, zen-bio, China) to determine fold enrichment. The comparative Ct method was used to analyze the data.

### Patient-derived xenograft (PDX) studies

To evaluate the effect of WNT7A in vivo, PDX studies were conducted. Four cases of fresh tumor tissues were obtained from patients undergoing surgery, which were provided by the First Affiliated Hospital of Fujian Medical University (Approval Number: FJMU-IACUC 2021-0299) and the written informed consent of each participant was obtained. The collected tumor tissues were cut into small fragments (2–3 mm in size) and placed in DMEM. Then 2–3 mm^3^ tumor blocks were implanted into the male nude mice (BALB/c nu/nu, 6–8 weeks old, Gempharmatech, China). When the primary mouse tumor grew to about 100 mm^3^, it was transplanted into the second generation. Two cases were chosen based on the second-generation expression of WNT7A, and transplanted into 6 mice of each group. Once the PDX tumors reach a suitable size or when the study endpoint is reached, the mice are euthanized, and the tumors are harvested for further analysis. Based on the formula below, we calculated the volume of the tumor: volume = length × width^2^ × 0.5.

### Statistical analysis

All data are presented as mean ± standard deviation (SD). GraphPad Prism 8.0 (GraphPad Software, San Diego, CA, USA) was used for statistical analysis and graphical data representation. Student’s *t*-tests, ANOVA, or χ2 tests were performed as appropriate to evaluate statistical significance. All experiments were performed in triplicate, and the data met the assumptions of the statistical analysis.

### Supplementary information

Supplemental Figure S1-S9 and Table S1-S4

## Data Availability

All the data associated with this study are available in the article and its Supplementary Information files and from the corresponding author upon reasonable request.
