# Supplementary material for: WNT7A promotes tumorigenesis of head and neck squamous cell carcinoma via activating FZD7/JAK1/STAT3 signaling
Source: Int J Oral Sci. 2024 Jan 22;16:7. doi: 10.1038/s41368-024-00279-y (PMC10800352; doi:10.1038/s41368-024-00279-y)
Supplement: Supplementary file 1 — Supplemental Figure S1-S9 and Table S1-S4 [file 41368_2024_279_MOESM1_ESM.pdf]

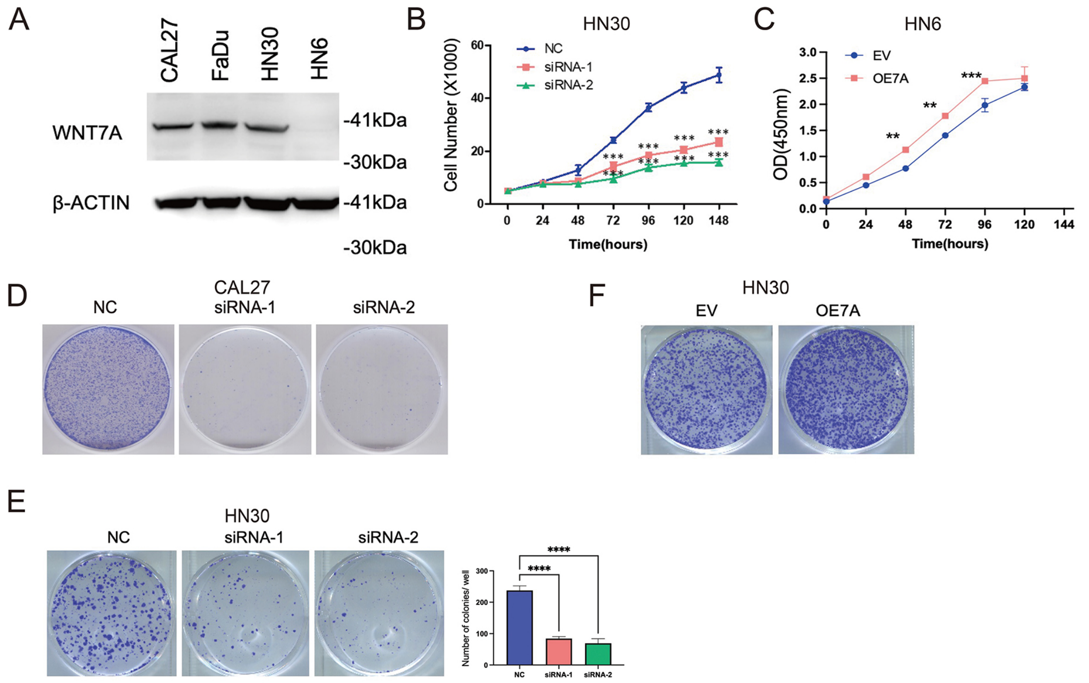

Supplementary Figure 1. Expression of WNT7A was positively associated with HNSCC cell proliferation. (A) Western blot analysis confirmed the WNT7A expression among HNSCC cell lines. (B) Knockdown of WNT7A significantly inhibited the growth of HN30 cells. (C) Overexpression of WNT7A significantly promoted the growth of and HN6 cells. (D, E) Colony formation assays revealed that knockdown of WNT7A inhibited the colony formation ability of CAL27 cells (D, related to Figure 2D) and HN30 cells (E). (F) Colony formation assays revealed that overexpression of WNT7A promoted the colony formation ability of HN30 cells (related to Figure 2F). Data is shown as mean ± SD (n = 3). *$p < 0.05$, **$p < 0.01$, ***$p < 0.001$, and ****$p < 0.0001$.

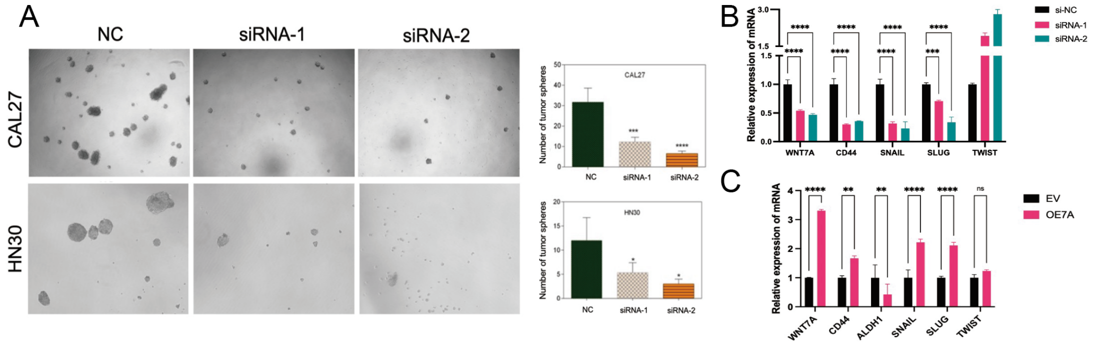

Supplementary Figure 2. Expression of WNT7A was positively associated with HNSCC cell self-renewal. (A) Knockdown WNT7A expression in CAL27 and HN30 cells correlated with decreased sphere formation. (B, C) Real-time RT-PCR demonstrated that WNT7A expression in HN30 (B) and HN6 (C) cells correlated with stemness markers. Data is shown as mean ± SD (n = 3). *p < 0.05, **p < 0.01, ***p < 0.001, and ****p < 0.0001.

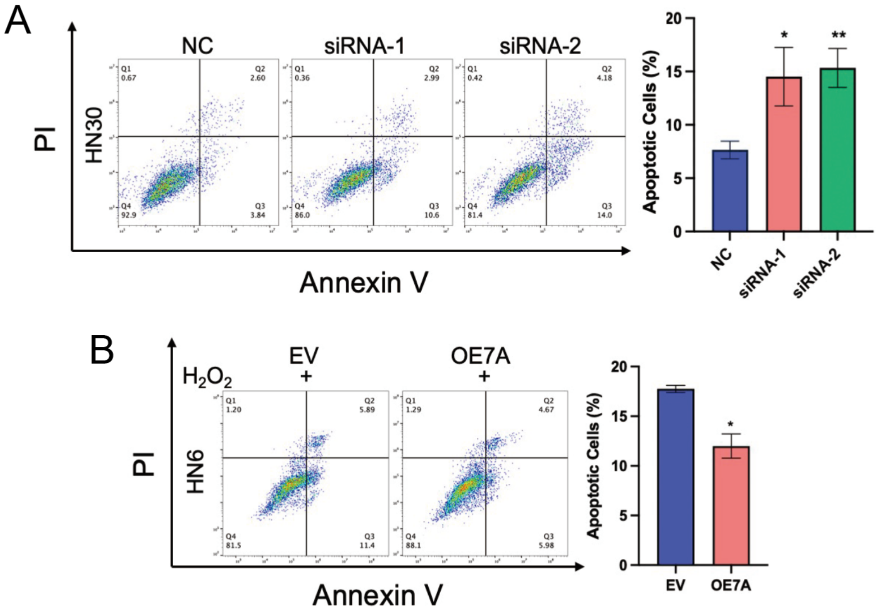

Supplementary Figure 3. Expression of WNT7A was positively associated with HNSCC cell anti-apoptosis. (A, B) Knockdown (A) and overexpression of WNT7A (B) in HNSCC cells positively correlated with anti-apoptosis ability. Data is shown as mean ± SD (n = 3). *p < 0.05, **p < 0.01.

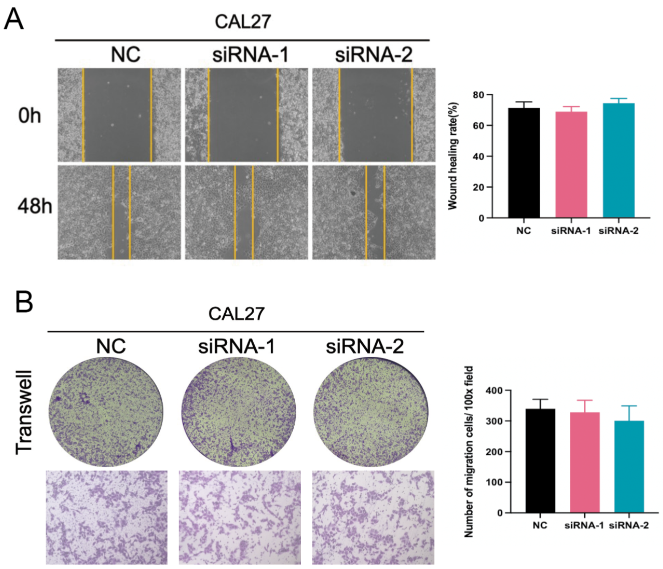

Supplementary Figure 4. Expression of WNT7A was not associated with HNSCC cell mobility. (A, B) Wound healing (A) and transwell assays (B) indicated no significant change in cell mobility following the knockdown of WNT7A expression in CAL27. Data is shown as mean ± SD (n = 3, p>0.05).

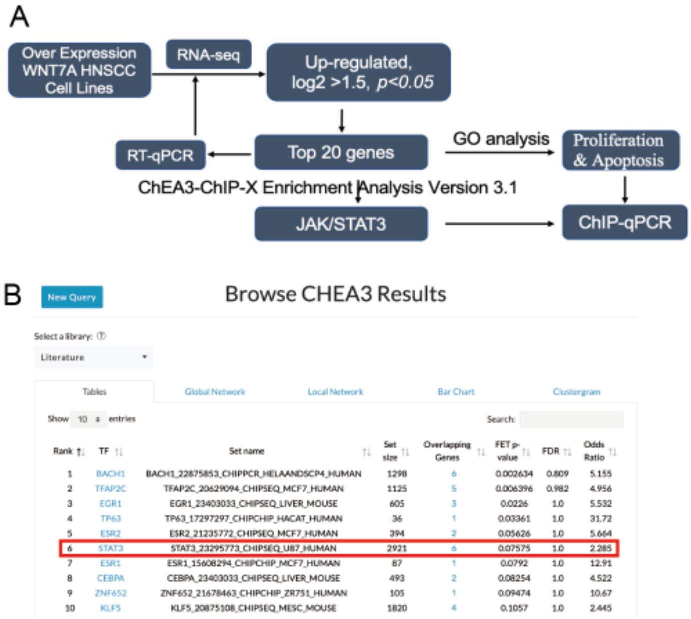

Rank	TF	Set name	Set size	Overlapping Genes	FET p-value	FDR	Odds Ratio
1	BACH1	BACH1_22875853_CHIPPCR_HELAANDSCP4_HUMAN	1298	6	0.002634	0.809	5.155
2	TFAP2C	TFAP2C_20629094_CHIPSEQ_MCF7_HUMAN	1125	5	0.006396	0.982	4.956
3	EGR1	EGR1_23403033_CHIPSEQ_LIVER_MOUSE	605	3	0.0226	1.0	5.532
4	TP63	TP63_17297297_CHIPCHIP_HACAT_HUMAN	36	1	0.03361	1.0	31.72
5	ESR2	ESR2_21235772_CHIPSEQ_MCF7_HUMAN	394	2	0.05626	1.0	5.664
6	STAT3	STAT3_23295773_CHIPSEQ_U87_HUMAN	2921	6	0.07575	1.0	2.285
7	ESR1	ESR1_15608294_CHIPCHIP_MCF7_HUMAN	87	1	0.0792	1.0	12.91
8	CEBPA	CEBPA_23403033_CHIPSEQ_LIVER_MOUSE	493	2	0.08254	1.0	4.522
9	ZNF652	ZNF652_21678463_CHIPCHIP_ZR751_HUMAN	105	1	0.09474	1.0	10.67
10	KLF5	KLF5_20875108_CHIPSEQ_MESC_MOUSE	1820	4	0.1057	1.0	2.445

Supplementary Figure 5. Exploration of downstream genes upregulated by WNT7A. (A) Flowchart of RNA-seq and informatics analysis to identify genes associated with WNT7A expression. (B) Prediction of transcription factors shows multiple up-regulated genes in RNA-seq analysis bind to transcription factor STAT3 by using ChEA3-ChIP-X Enrichment Analysis Version 3.1.

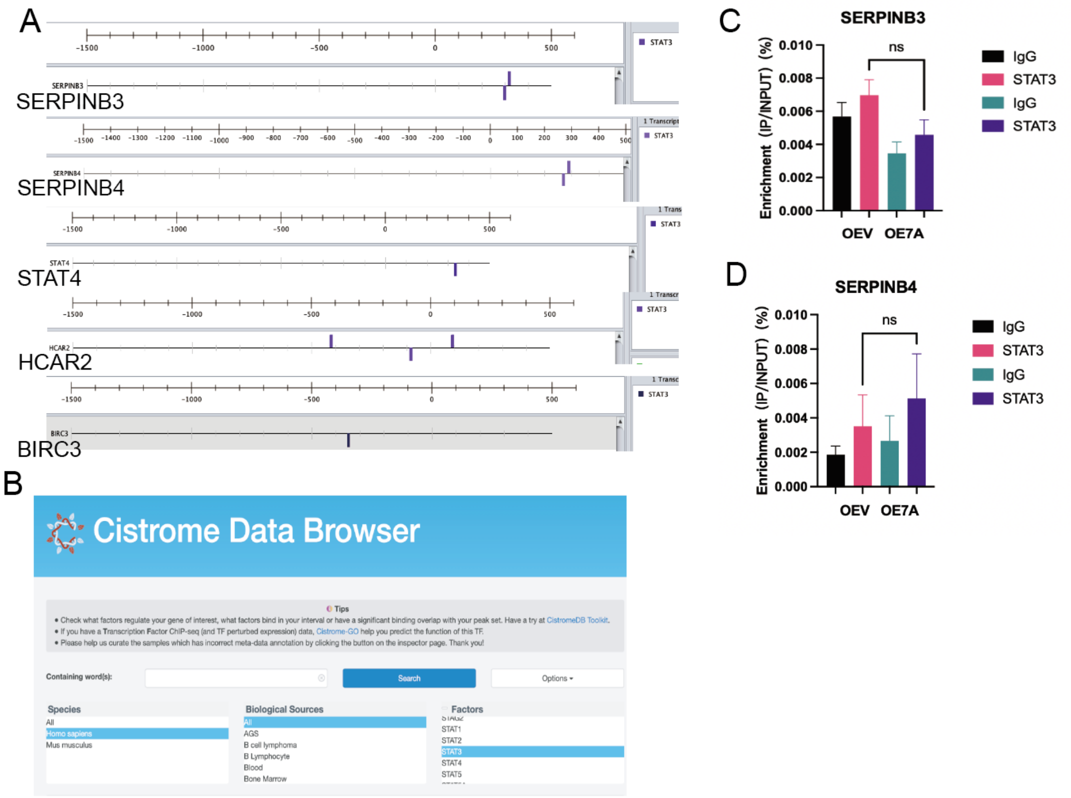

Supplementary Figure 6. WNT7A upregulated expression of STAT3 target genes in HNSCC cells. (A) The predicted transcription factor binding sites of these genes by using CiiiDER2. (B) Design of ChIP-qPCR primers by using Cistrome Data Browser. (C, D) ChIP-qPCR observed that SERPINB3 and SERPINB4 were not identified as direct target genes of STAT3 in HNSCC cells. Data is shown as mean ± SD (n = 3). ns, p > 0.05.

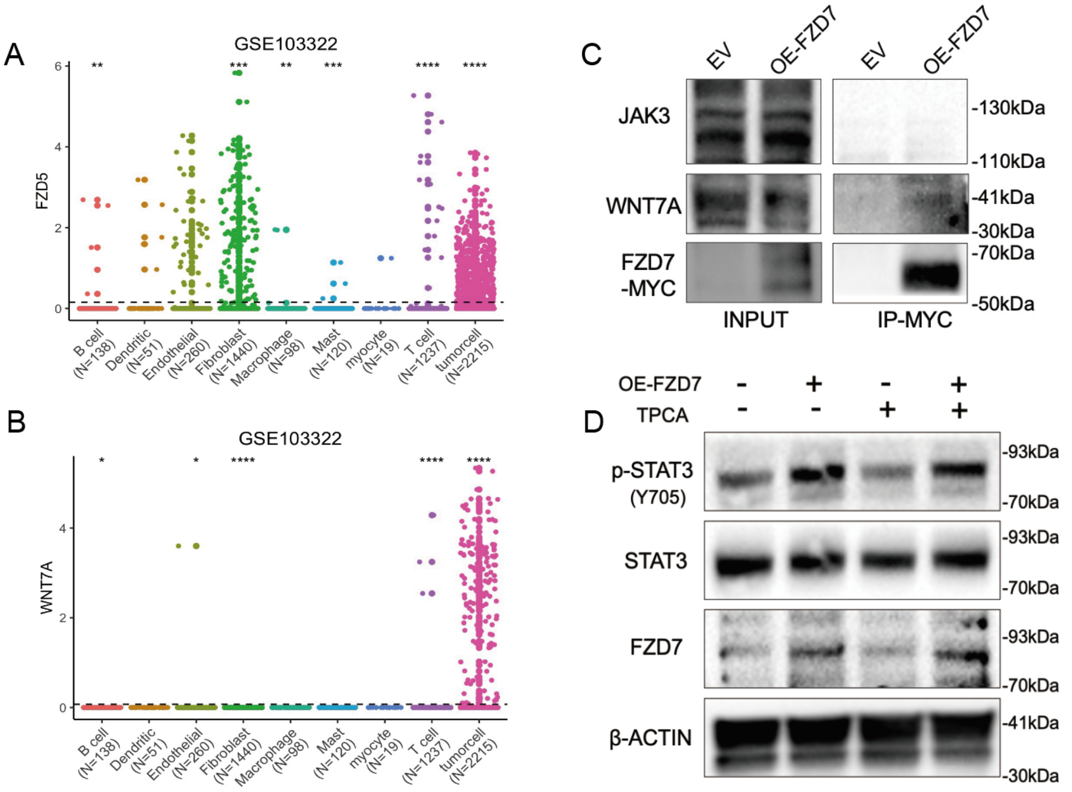

Supplementary Figure 7. WNT7A may activated STAT3 signaling pathway through FZD7/JAK1. (A, B) Analysis of single-cell sequencing data (GSE103322) reveals FZD5 mainly expressed in fibroblast, T cell and tumor cell (A), while WNT7A mainly enriched in cancer cell (B). (C) Co-immunoprecipitation results found no interactions between FZD7 and JAK3. (D) Elevated FZD7 expression increased pSTAT3 Y705 levels but decreased after TPCA treatment.

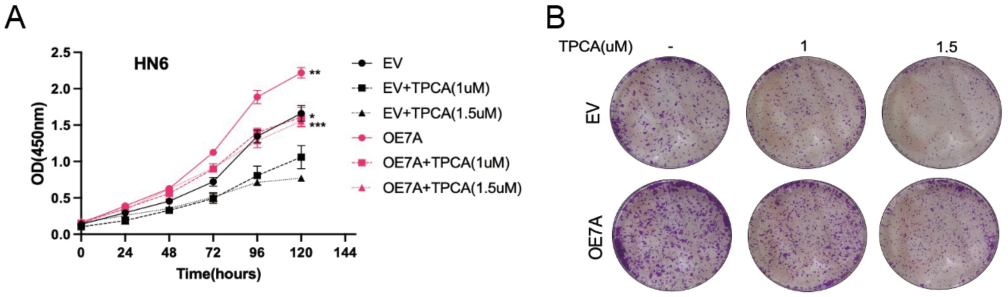

Supplementary Figure 8. TPCA inhibited WNT7A-induced activation of STAT3 signaling and proliferation in HNSCC cells

(A) CCK-8 assay shows that TPCA treatment decreased the cell proliferation promoted by WNT7A overexpression in HN6 cells. (B) Colony formation assay shows that TPCA treatment impaired the colony formation ability of HN30 cells (related to Figure 7D). Data is shown as mean ± SD (n = 3). *p < 0.05, **p < 0.01, and ***p < 0.001.

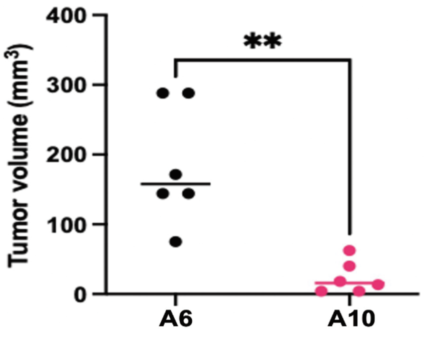

Supplementary Figure 9. A comparison was made between the tumor size of A6 (n=6) and A10 (n=6). A10 displayed a smaller tumor volume when compared to A6 (related to Figure 8). **, p<0.01.

Supplementary Table 1.

The sequences of siRNAs used in the transfection experiments.

Name	Sense（5'-3'）	Antisense（5'-3'）
NC	UUCUCCGAACGUGUCACGUTT	ACGUGACACGUUCGGAGAATT
WNT7A-siRNA-1	CCUGGACGAGUGUCAGUUUTT	AAACUGACACUCGUCCAGGTT
WNT7A-siRNA-2	GGAGAACAUGAAGCUGGAATT	UUCCAGCUUCAUGUUCUCCTT
WNT7A-siRNA-3	CCACCUUCCUGAAGAUCAATT	UUGAUCUUCAGGAAGGUGGTT

Supplementary Table 2.

The primer sequences of the RT-qPCR.

Gene	Sense（5'-3'）	Antisense（5'-3'）
WNT2B(homo)	CTCATCAGCAGGGGTAGTCC	AAAACGGACACCGTAGTGGA
WNT3A(homo)	TGTTGGGCCACAGTATTCCT	ATGAGCGTGTCACTGCAAAG
WNT4(homo)	CCTTCGTGTACGCCATCTCT	GCCTCATTGTTGTGGAGGTT
WNT5A(homo)	CCACATGCAGTACATCGGAG	CACTCTCGTAGGAGCCCTTG
WNT7A(homo)	AGTACAACGAGGCCGTTCAC	GCACGTGTTGCACTTGACAT
WNT7B(homo)	AAGCTCGGAGCACTGTCATC	CCCTCGGCTTGGTTGTAGTA
WNT10A(homo)	AATGCCAACACCAATTCAGG	CAACTCGGTTGTTGTGAAGC
WNT10B(homo)	GCAAGAGTTTCCCCCACTCT	GATTGCGGTTGTGGGTATC
CD44(homo)	CTGCCGCTTTGCAGGTGTA	CATTGTGGGCAAGGTGCTATT
ALDH1(homo)	TTGAGCGGGCTAAGAAGTAT	CCTCCTCCACATTCCAGTTT
SNAIL(homo)	CGGAAGCCTAACTACAGCGA	GCCAGGACAGAGTCCCAGAT
SLUG(homo)	TCAAGGACACATTAGAACTCACAC	CTACACAGCAGCCAGATTCC
TWIST(homo)	TACGCCTTCTCGGTCTGGAG	TTCTCTGGAAACAATGACATCTAGG
SERPINB3(homo)	GCCACCGCTGTAGTAGGATT	TGCCATAGAAGAGGATGCTGTT
SERPINB4(homo)	GCCACCGCTGTAGTAGTAGT	TGCCATAGAAGAGGATGCTG
STAT4(homo)	AAGCCATCTCGGAGGAATAACT	TTGTAGTCTCGCAGGATGTCA
HCAR2(homo)	GGACAACTATGTGAGGCGTTGG	GATGATGGCTGCTGTCCGATT
BIRC3(homo)	GATGGTGGACTCAGGTGTTG	GGCTTGAACTTGACGGATGA

Supplementary Table 3.

The primer sequences of the ChIP-qPCR.

Gene	Sense（5'-3'）	Antisense（5'-3'）
IRF1(homo)	CACGTCTTGCCTCGACTAAGG	AGTGTTTGGATTGCTCGGTG
HCAR2(homo)	CTAGTGAATGCTCCAGCAAGG	GACATGACATAAAGGCAGGCGT
BIRC3(homo)	TCATGGAAATCCCCGAGTGG	AGGGGAACTCCAGCGGTAAT
STAT4(homo)	TCTCCTCCCACTTGAGGCTT	GGCAGTTTCTGCGTGTTAGC
SERPINB3(homo)	AGACCTCTTTCCAGTAATTTCAACC	GGAGTGTCCCAGACCCTAACA
SERPINB4(homo)	CATATCCTGGTGTGTGAGACTGGA	GGAGCCAATGAAGTTGGTGTATG

Supplementary Table 4.

The antibodies used in protein-related experiments.

Antibody	Item No	company
WNT7A Rabbit mAb	ab274321	Abcam
Frizzled 7 Rabbit mAb	ab64636	Abcam
Frizzled 7 Rat mAb	151143	Thermo Fisher Scientific
Snail (C15D3) Rabbit mAb	3879S	Cell Signaling Technology
Slug (C19G7) Rabbit mAb	9585S	Cell Signaling Technology
Stat3 (D3Z2G) Rabbit mAb	12640S	Cell Signaling Technology
Phospho-Stat3 (Tyr705) (D3A7)Rabbit mAb	9145S	Cell Signaling Technology
Jak1 (E3A6M) Rabbit mAb	29261S	Cell Signaling Technology
Phospho-Jak1(Tyr1034/1035) (D7N4Z) Rabbit mAb	74129S	Cell Signaling Technology
Jak3 (D1H3) Rabbit mAb	8827S	Cell Signaling Technology
Phospho-Jak3 (Tyr980/981) (D44E3) Rabbit mAb	5031S	Cell Signaling Technology
β-Catenin (D10A8) Rabbit mAb	8480S	Cell Signaling Technology
Phospho-β-Catenin (Thr41/Ser45) Rabbit mAb	9565S	Cell Signaling Technology
β-Actin (8H10D10) Mouse mAb	3700S	Cell Signaling Technology
α-Tubulin (11H10) Rabbit mAb	2125S	Cell Signaling Technology
Lamin A/C (4C11) Mouse mAb	4777S	Cell Signaling Technology
Cleaved Caspase-3 (Asp175) (5A1E) Rabbit mAb	9664S	Cell Signaling Technology
Goat anti-Rat IgG (H+L) Cross-Adsorbed Secondary Antibody, Alexa Fluor™ 555	A-21434	Thermo Fisher Scientific
Anti-rabbit IgG (H+L), F(ab')2 Fragment (Alexa Fluor® 488 Conjugate)	4412S	Cell Signaling Technology
Anti-rabbit IgG (H+L), F(ab')2 Fragment (Alexa Fluor® 594 Conjugate)	8890S	Cell Signaling Technology
Anti-Flag Tag Antibody (Monoclonal, 1E6)	M30971	Boster Biological Technology
Anti-Myc Tag Antibody (Monoclonal, 10D11)	M00026-2	Boster Biological Technology
GAPDH Rabbit Monoclonal Antibody	M00227	Boster Biological Technology
HRP Conjugated AffiniPure Goat Anti-Rabbit IgG (H+L)	BA1054	Boster Biological Technology
HRP Conjugated AffiniPure Goat Anti-Mouse IgG (H+L)	BA1050	Boster Biological Technology
